# An Exploration of the Psychological Traits Deemed Crucial for Success in UK Special Forces Operators

**DOI:** 10.3390/bs15091194

**Published:** 2025-09-01

**Authors:** Shane Breen, Stewart Cotterill

**Affiliations:** Centre for Human Performance Research, Health Sciences University, Bournemouth BH5 2DF, UK; shanebreen7@hotmail.com

**Keywords:** special forces, performance, development, mindset

## Abstract

Special forces operators are increasingly being utilized as the weapon of choice by many governments on the geopolitical stage. Given the specialized and high-risk nature of special forces operations, it is important to understand the differences that exist when comparing the psychological traits of these groups to regular military forces. An understanding of these traits is crucial when looking to select, develop, and support the most appropriate individuals to succeed in these roles. While previous research has painted a clear picture relating to personality differences between special forces operators and the wider military forces, there is still little research that has explored the psychological traits that both influence and determine performance. As a result, the aim of this study was to explore the perceptions of former United Kingdom (UK) special forces operators regarding the psychological traits they believed were crucial for success as a special forces operator in the UK military. Participants in this study were 20 former UK special forces operators, each having transitioned from active service to civilian life within the previous five years. Data were collected and analyzed using reflexive thematic analysis. Results suggested a specific profile of UK special forces operators composed of nine specific factors: resilience, adaptability, self-belief, perseverance, emotional regulation, self-control, drive, humility, and stubbornness. With the last two relatively novel compared with relevant research in similar populations. These findings can help to underpin the development of special forces-specific programs of support and development.

## 1. Introduction

The Special Operations Forces (SOFs) component of national military forces plays a crucial role in global geopolitics, undertaking high-stakes missions that have significant national and international implications. These special forces are selected and trained to deploy in small numbers and often tackle operations beyond the capabilities of standard military units (Robinson, 2013), actively contributing to strategic decision-making and facilitating the projection of national power and influence. Resulting in the success of these specialist units extends beyond military considerations and bears geopolitical significance (Jones, 2023).

The nature of the roles these SOFs undertake is often distinctly different from conventional military forces. In this context, special military operations have been characterized as “encompassing the use of small units in direct or indirect military actions focused on strategic or operational objectives. These actions require units with combinations of specialized personnel, equipment, and tactics that exceed the routine capabilities of conventional military forces” (Joint Special Operations University (U.S.) & Center for Special Operations Studies and Research, 2015, p. 1). These SOFs can be deployed across the broad spectrum of military operations, often with very little notice given and, at times, little specific preparation. As such, they can be viewed as “specialist generalists”, retained in high demand by policymakers and with significant value placed upon what can be described as their adaptive expertise (Mees et al., 2020). It is also important to distinguish between elite and special forces. Searle (2017) suggested that an elite unit is very good at what they do (highly proficient at a specific task). Hence, they are “elite”, but they are not necessarily special. “Special”, on the other hand, implies different (in the context of conventional military operations), rather than merely “better” (Searle, 2017, p. 11). As such, special forces can be elite but are not elite by definition.

In recent years, there has been rapid growth in SOFs in industrial democracies, both in terms of numbers and resources, driven by a significant growth in the number and priority of missions assigned to them (Shamir & Ben-Ali, 2017). SOFs possess extremely varied and at times tension-filled abilities for action in a variety of fields of operation straddling high- and low-intensity engagements, nation-building, humanitarian missions, training indigenous forces, and liaising with other national forces (Fernando, 2013). In this context SOF can be described as units trained to operate in small teams, behind enemy lines, utilizing a wide range of organizational resources and special capabilities that are employed to provide innovative solutions to problematic circumstances (Marquis, 1996).

In undertaking these operations, these special forces units are exposed to an extreme range of psychological and physical pressures that are suggested to be much more than those encountered by conventional military personnel or the civilian population (O’Hara et al., 2022). Given the specialized and high-risk nature of special forces operations, it is important to understand what distinguishes those individuals who are successful in their roles in terms of psychological traits from other military personnel. Such an understanding could inform recruitment processes, training directives, mental health support systems, and even geopolitical policies, thereby enhancing the preparedness and resilience of these invaluable teams (Anglim, 2020; Skoglund et al., 2020). While there is extensive research focused on conventional military forces, there is much less focused on special forces (Ytterbøl et al., 2022). Indeed, much military-focused research does not distinguish between differences in tactical military personnel, their roles, levels of training, or expertise (Raabe et al., 2021), so the special characteristics of SOFs may go largely unnoticed or unaddressed (Ytterbøl et al., 2022).

Of interest in seeking to understand SOFs are the psychological traits, characteristics, and behaviors that mark them as potentially different from regular military forces. These psychological traits have been suggested to be essential determinants of human performance, especially in high-stress and high-risk situations like those encountered by special forces operators (Laureys et al., 2023).

Psychological traits in this context can be defined as “dimensions of individual differences in tendencies to show consistent patterns of thoughts, feelings, and actions” (McCrae & Costa, 2003, p. 25). The terms “psychological” and “personality traits” are often used interchangeably in the psychology literature to refer to stable dimensions of the individual. In recent years there has been a prioritization of the Big Five Model of personality (openness, conscientiousness, extroversion, agreeableness, and neuroticism) in military-focused research.

Several studies have explored the personality traits of special forces operators utilizing the Big Five Model of personality. For example, D. E. Braun et al. (1994) compared the personality characteristics of 139 U.S. Navy SEAL (SEa Air Land) operators to the broader U.S. population. The comparison was based on the Big Five personality dimensions. The authors reported that the SEALs exhibited lower scores for neuroticism and agreeableness compared to civilians, while their scores for conscientiousness and extraversion were higher. D. E. Braun et al. also reported that SEALs with more experience exhibited higher levels of conscientiousness and lower extraversion compared to their less experienced counterparts. Commissioned officers (individuals holding a commissioned officer position) were found to have higher scores on both of these dimensions in contrast to enlisted operators. In a similar study based on the Big Five personality traits, Skoglund et al. (2020) reported comparable results among Norwegian (NORSOF) special forces operators. Their results demonstrated that younger personnel in the NORSOF scored significantly lower on emotional stability scores than their older colleagues, and that those without any combat deployment experience scored higher on agreeableness and slightly lower on emotional stability. In this study, NORSOF officers also reported higher extroversion. Based on these results, a personality profile of the operator emerged characterized by lower extraversion and agreeableness and higher emotional stability related to basic forces. Emotional stability has been highlighted as being important for high-risk operational personnel (Skoglund et al., 2020). In addition, Huijzer et al. (2022) reported that special forces commandos in the Netherlands were less neurotic and more conscientious compared to the general public. Bech et al. (2021) also examined the personality traits of 32 male candidates who completed an eight-month intensive training program to become operators in the Danish Naval Special Warfare Group (also known as Frogmen). Reporting that Frogman candidates had significantly higher levels of conscientiousness and agreeableness than the university students. Relative to the university students, the Frogmen showed higher scores in extraversion and lower scores in neuroticism at the end of training compared to the start. Whilst this research offers an interesting insight into personality and the nature of special forces operators, these studies do not explicitly explore the psychological traits underpinning performance in this context. Indeed, while interesting, the Big Five approach has been criticized as being restrictive, as many of the most interesting psychological trait-like characteristics are thought to lie beyond the boundaries of the Big Five Model (Bainbridge et al., 2022), suggesting the Big Five Model can be too reductive in seeking to understand psychological traits in the applied context.

Previous researchers have also sought to understand the mindset of special forces operators. In the broader sense, mindset can be viewed as a set of situational, cognitive processes that are called forth by a given task (Gollwitzer, 2012). In seeking to understand the mindset of special operations forces in Denmark, Dalgaard-Nieslen and Holm (2018) suggested a framework organized into three areas: tactical proficiency (TP), organizing, and coping with complexity (CWC). For TP, the authors listed self-assured, self-reliant, physically strong, psychologically stable, focused, rigorously selected and trained, and risk tolerant. In conceptualizing “organizing”, the authors suggested informal, innovative, delegating, non-hierarchical, disregard for admin, flexible, loyal, arrogant, collaborating, competitive, and rule-breaking. CWC was characterized as being culturally aware, creative, analytical, and adopting cross-disciplinary approaches. In addition, E. N. Smith et al. (2020) considered the mindset of US Navy SEALs, suggesting that the mindset of the operator is important as it can impact upon the individual’s performance in stressful situations. E. N. Smith et al. (2020) specifically explored stress mindsets, suggesting a “stress is enhancing” mindset rather than a “stress is debilitating” mindset was crucial for operator success. Understanding mindset is important, as it interacts with the underpinning psychological traits to determine the nature of the individual operator.

While there is limited research focused on psychological traits in special forces operators, researchers have explored these factors in regular military forces. For instance, Bartone et al. (2008) found that psychological hardiness was a vital determinant of success amongst candidates for the U.S. Army, establishing a foundation of a soldier’s psychological readiness to engage and overcome difficulties under dangerous conditions. Oral and Karakurt (2022) support this perspective, suggesting that hardiness is important as it reflects the individual’s capacity to withstand stress, uncertainty, and adversity.

Understanding risk-taking behavior appears to be another important consideration when exploring military populations, and potentially for SOFs. A four-factor model offering a psychological framework for understanding soldier behaviors in high-risk scenarios such as combat was proposed by Prykhodko et al. (2021). The model identifies key attributes and qualities crucial for maintaining optimal performance in demanding circumstances like battles. These attributes include the ability for volitional effort, military brotherhood, professional identity, and self-control. Central to this model are further attributes such as strong willpower, patriotism, and personal adaptive resources. These additional components were suggested as critical factors influencing soldiers’ efficacy in battle (Prykhodko et al., 2021). However, while all these factors are indeed relevant in a combat setting, the model primarily focuses on the mental preparedness and willingness of soldiers to face risks during combat deployment. It does not specifically outline any psychological traits inherent to special forces personnel, instead focusing on the wider conventional military. Developing an understanding of traits specific to SOFs might be particularly relevant when considering the selection, training, and development of these operators.

Understanding the psychological traits of SOFs is crucial in seeking to select, develop, and support the most appropriate individuals to excel in these roles. While previous research has painted a clear picture relating to personality and mindset differences between special forces operators and the wider military forces, there is still little research that has explored the psychological traits that both influence and determine performance in this population.

It is also important to note that not all special forces are the same. While national governments might have the same strategic priorities for their special forces provision, these needs are often met in different ways between SOFs. For example, the available palette of SOFs is considerably larger for the USA compared with the UK (Allwood, 2022). A fact that implies that the personnel needed may differ depending on the specialist or generalist demands placed upon the group. As such, research undertaken on one population in relation to psychological traits for success might not be applicable in another national military context. While there will be similarities, there could also be differences. For example, in the UK context the Special Air Service regiment (SAS) and the Royal Marines’ Special Boat Service (SBS) units are similar in organization, equipment, and personnel selection procedures, and also perform the same broad range of tasks while each retaining some specialist capabilities—with the SAS specializing in land and airborne operations and the SBS in maritime and littoral operations (Anglim, 2020). This suggests that there could also be differences in SOFs within the same national context. While both the SAS and SBS have the same selection procedures, the psychological traits needed for longevity of success might be different.

There also appear to be changes in the personality of operators post selection. Studies by D. E. Braun et al. (1994) and Skoglund et al. (2020) on SOFs indicated that age and experience might be factors in psychological trait change. The D. E. Braun et al. (1994) study showed differences with regard to age, with more experienced SEALs showing higher scores on conscientiousness and lower scores on extraversion than the less experienced. The study by Skoglund et al. (2020) reported differences within the NORSOF operators: compared to older operators, younger operators showed lower scores on emotional stability; those without combat-deployment experience showed higher scores on agreeableness and lower scores on emotional stability than those with such experience; and operators with officer ranks showed higher scores on extraversion than specialists. These results suggest that what you need to get into the service is not necessarily what you need to have longevity and to excel in the role. Indeed, while special operations personnel are carefully selected and trained and therefore anticipated to be prepared and able to cope well, there exists the potential for significant “wear and tear” on operators and families, particularly based on the nature and number of deployments they perform (Ogle & Young, 2016). Suggesting the psychological traits required to cope with the reality of being a special forces operator might change over time. As a result, the aim of this study was to explore the perceptions of former (veteran) SOF service personnel who had significant longevity in their careers regarding the psychological traits to be successful as a UK special forces operator.

## 2. Method

### 2.1. Methodology

This study adopted a qualitative methodology, specifically utilizing reflexive thematic analysis. Advocated for its versatility, reflexive thematic analysis offers a robust lens to examine, dissect, and document patterns or themes embedded in data (V. Braun & Clarke, 2021). The approach involves systematically organizing and interpreting data to identify recurring themes or patterns of meaning (Roberts et al., 2019). This research adopted this method to explore the perceptions of former special forces operators regarding the psychological traits required for success.

### 2.2. Participants

Participants in this study were 20 former (veteran) UK special forces operators, each having transitioned from active service to civilian life within the previous five years. Participants were male, with an average age of 40.5 years, an average military service time of 21 years, and an average service time in the UK special forces of 16.6 years. The eligibility criteria for participants included being no more than five years removed from service. These criteria were applied to ensure the presence and reliability of a current special forces mindset, which could otherwise be potentially diluted by prolonged exposure to civilian life (Reyna et al., 2016). Participants were recruited through personal contact by the authors, one of whom is also a member of the veteran SOF community. Participants were recruited for this study due to their long experience as operators and the pragmatic challenges of recruiting currently active operators in the UK.

### 2.3. Procedure

After institutional ethical approval was gained and informed consent was obtained from all participants, each person received a unique identifying code to ensure anonymity, ranging from Operator 1 to Operator 20. Subsequently, individual semi-structured interviews were conducted. These interviews were conducted using Microsoft Teams video calls due to the geographical dispersion of participants. The decision to utilize video conferencing was a purposeful one to increase the ability to recruit participants and to offer flexibility in scheduling for these participants (Keen et al., 2022). Moreover, this video call platform allowed for seamless audio recording of the interview sessions, ensuring the fidelity of the data collected.

Each interview ranged in length from 30 to 75 min (M = 42 min), depending on the depth and breadth of the participants’ responses. During these interviews, participants were asked to respond to a series of open-ended questions. These questions were designed to elicit detailed insights about their perceptions of the psychological traits required to be successful in service (Wildemuth, 2017). The interview questions were open-ended by design, a recommended practice in reflexive thematic analysis (Nowell et al., 2017), to allow participants to freely discuss their experiences and observations about required psychological traits. Examples of interview questions in this study include, as part of the interview schedule, “In your opinion what are the psychological traits shared by Special Forces soldiers?” and “Do you believe that a particular mindset or psychological approach impacts upon success within the Special Forces? If so, can you describe this mindset?” At the start of each interview, psychological traits in the context of the study were defined to provide clarity to participants.

To guarantee accurate and comprehensive data capture, the interviews were recorded and transcribed. The process of transcription ensures that the entirety of the participants’ responses was preserved for subsequent analysis (Jamshed, 2014). Additionally, the interviewer took notes throughout each session. These notes served as a supplementary record and helped capture non-verbal cues and other pertinent details that could contribute to the overall understanding of participants’ responses (Sutton & Austin, 2015). This multi-pronged approach to data capture ensured a robust and reliable foundation for the ensuing data analysis.

## 3. Data Analysis

Data gained from the interviews were analyzed using reflexive thematic analysis. This form of data analysis aims to organize and describe the data through identifying patterns, also known as themes, and similarities and/or differences that occur across the data set (V. Braun & Clarke, 2019). Thematic analysis offered the researcher the opportunity to interpret and describe both the data of each individual participant and the whole data set (Sparkes & Smith, 2014). For the purpose of this study, the researchers followed V. Braun and Clarke’s (2021) six-step method to ensure that a clear process and structure for the analysis and interpretation of the data was adopted. While these steps are sequential, the process was less linear, with the authors returning to different stages in an iterative process based upon the data.

The first step involved each interview being transcribed verbatim, this step enabled the researchers to familiarize themselves with the data. Through subsequent reading and re-reading of the transcripts, a comprehensive understanding of each person’s account was developed. Second, systematic coding was employed on each transcript; material of interest or significance to the research question was identified through initial notes made in one of the margins of the transcript and the highlighting of relevant associated text. The third step outlined in the process was to acknowledge and note down any resultant themes conceptualized from each transcript and the overall data set. The fourth step of analysis focused on reviewing the themes to ensure that each one accurately reflected both the coded extracts and the data set as a whole. Naming and defining each theme to clearly represent its data content marked the penultimate stage of analysis. The sixth and final step involved producing a report, selecting quotes and extracts from the data that accurately represented each theme, and relating the analysis back to the research question and the associated literature.

### Rigor

Ensuring the quality of the qualitative data was pivotal given the data’s inherent subjectivity. Credibility was established through a deep engagement with the data, ensuring that interpreted themes genuinely mirrored participants’ experiences, as highlighted by B. Smith and McGannon (2018). The richness of the data was achieved using open-ended questions, ensuring a detailed capture of participants’ experiences. Throughout the study, given the first author’s previous background in the military, there was a heightened self-awareness regarding potential biases and interpretive pitfalls. By rigorously upholding ethical standards, including participant consent and data security, and maintaining continuous dialogue with the second author, the first author ensured interpretations remained genuine, credible, and confirmable to the data (Šimundić, 2013). Adherence to the principles outlined by Tracy (2010) further strengthened the study’s logical coherence and resonance with the audience; steps included the use of journaling by the first author as well as member reflections (B. Smith & McGannon, 2018). This comprehensive approach provides greater credibility to the findings, emphasizing their rigor and reliability.

## 4. Results

The analysis of these data resulted in the conceptualization of ten primary themes: resilience, adaptability, self-belief, perseverance, emotional regulation, humility, drive, self-control, stubbornness, and coping mechanisms. These themes have been used as a foundation for the subsequent results and discussion sections.

### 4.1. Resilience

A significant finding from the interview data was the importance of resilience in the context of special forces operations. Various participants identified resilience as an essential trait, as articulated by Operator 4, who reflected that “I’d say we [special forces operators] are very, very mentally resilient, hardy lads, and this helps us, you know, adapt to changing and challenging situations”. This reflection illustrates the mutual reinforcement and interchangeability of the use of resilience and hardiness from the perspective of the operators in this study.

Interestingly, some operators suggested their resilience was developed through their early life experiences. These participants believed that these formative experiences, such as moving frequently during childhood, forced them to develop a “thick skin” and to become more resilient. This view was further supported by Operator 11 who reflected that “Moving a lot as a child... It built an ability to accept changing environments and just crack on [continue] regardless of the hardships experienced”.

### 4.2. Adaptability

The degree to which participants could evolve and adapt their approach based upon the situation was highlighted by numerous participants as being crucial. This adaptability was seen to be crucial to the participant’s professional functionality, shaped by the highly fluid, multifaceted, and demanding nature of their military assignments. The importance of adaptability was highlighted by operator 16 who stated the following:

During ops [special forces operations/missions], plans can change in a split second. As they say “no plan survives the first contact” [with the enemy]. You’ve got to be quick to adjust your strategies, behaviors, even your attitudes.

Participants in this study also highlighted the link between adaptability and resilience, suggesting that one could positively impact the other. This view was exemplified by participant fifteen, who reflected the following:

This adaptability, I’ve found, not only helped me handle the immediate situation but also reduced the overall stress and let me bounce back quicker from the unexpected. That made me more resilient to the shit thrown at me.

### 4.3. Self-Belief

The importance of self-belief was something consistently reported by participants. Operators indicated that possessing belief in their own abilities gave them the fortitude to persevere during demanding missions and strenuous selection processes. For example, Operator 9 explained the following:

In the toughest moments, it’s your self-belief that keeps you going. It fuels your grit. It’s like an inner voice that tells you that you can keep trucking on, that you can complete the task, no matter how hard it might be.

This view was supported by Operator 2, who stated that “Badged guys [members of the special forces] have that self-belief. And on selection if you don’t have it, you simply won’t keep going, you won’t pass”. This view of the importance of self-belief was corroborated by numerous other operators who similarly connected a lack of self-belief and the presence of self-doubt to unsuccessful attempts during the selection process. Operator 7 reinforced this view, reflecting the following:

Many of the guys who didn’t make it through selection seemed to lack full belief in their own capabilities. They tended to second-guess themselves, which led them to believe they were falling short of the DS’s [Directing Staff on selection] expectations. Ultimately, this lack of self-belief appeared to erode their perseverance.

### 4.4. Perseverance

Perseverance was another dominant theme identified by the operators as critical to their role in the SOFs. The operators often tied this trait directly to the successful completion of rigorous training and demanding missions. The importance of perseverance was highlighted by participant nine, who stated the following:

Sometimes you just have to keep going, it is important no matter what, to keep going, to not give in. Yes, it can get really tough and might seem impossible, but you have to trust in yourself and the rest of the boys to make it to the end.

The operators saw a close relationship between self-belief and perseverance, where each trait appeared to reinforce and strengthen the other, as articulated by Operator 15, “Your self-belief is the foundation for your perseverance. The more you trust in your own abilities, the more you’ll be willing to push through challenges and keep moving forward”. This view suggests that the interrelated nature of many of these highlighted traits is a crucial part of understanding the psychological traits of SOFs.

### 4.5. Emotional Regulation

The ability to manage and regulate emotions emerged as a significant theme from the interviews. Several operators highlighted its importance, particularly within the context of the high-stress, rapidly changing environments they often operate in. Participants emphasized how effective management of emotions can be a direct determinant of mission success while on operations. For example, Operator 16 highlighted this point, noting the following:

During operations, emotions can run high, and it can be easy to let them get the better of you. But, maintaining control over your emotions, not letting stress or fear dictate your actions, is vital. When you can regulate your emotions, you make clear-headed decisions, and your performance won’t suffer.

Furthermore, several operators emphasized the significance of emotional regulation beyond high-stakes mission scenarios. The participants highlighted the importance of emotional regulation in managing interpersonal relationships within the service as well. For example, Operator 5 stated the following:

Even when you’re not on ops [operations], emotional regulation plays a big role. Sometimes dealing with dickheads back at base can be as hard as the operations themselves. Keeping your emotions in check, understanding how and when to gob off [speak up] and when to wind your neck in [say nothing/back down from a disagreement] can help maintain synergy among the lads.

### 4.6. Humility

Humility emerged as an important trait in the narratives of the operators interviewed in this study. Participants detailed that humility empowered them to continuously evolve and adapt by learning from others, particularly those with more experience. As stated by Operator 8:

The moment you think you know it all, you’ve set yourself up for failure. We’ve got to stay humble, always ready to learn and grow. The most experienced among us, they’ve got a wealth of knowledge to share if you’ve got the humility to listen and learn.

The presence of this trait appears to ensure an openness to exploring different perspectives, methods, and strategies. The ability to approach tasks without ego, recognizing that there is always room for improvement, and the willingness to change tactics based on new information were seen as vital for success. This view was articulated by Operator 14, who shared the following:

The dynamics of our operations are complex and ever-changing. Stubbornly clinging to your way of doing things can be detrimental. Humility helps to keep an open mind, ready to adapt. Just look at them up the road [referring to a different unit within special forces], not changing CQC [close quarter combat] drills in years even though all other tier one [elite] units have.

### 4.7. Drive

Drive featured prominently during the interview process, particularly in relation to the driving forces that initially propelled operators towards special forces selection. A range of intrinsic motivations that sparked their interest in joining the special forces in the first place were discussed. For instance, Operator 3 shared the following:

I always had a fascination for special forces after hearing dits [stories] about what they get up to, and there is that mystery surrounding what special forces does. But more importantly, I wanted to challenge myself, to see if I could measure up.

Furthermore, a notable trend from some operators was the expressed desire for feedback as a form of their drive and motivation. Participants described the satisfaction derived from passing selection or topping a course as a tangible reward for their dedication and hard work. In this context, Operator 1 stated the following:

It’s not just about being told you’re good at what you do. It’s about proving it to yourself and seeing the results of your effort, like getting through the selection process or outperforming others on a course. That’s the real payoff for me.

This concept of being driven (participants’ term for motivation) was outlined by participants as being an important characteristic of SOFs and required to maintain success over an extended period.

### 4.8. Self-Control

The importance of self-control was a consistent theme among the participants. All twenty operators attested to the critical role this trait plays in successful special forces operations. Participants shared a unanimous view that rigorous preparation and continuous practice enhanced their confidence and honed their professional skills, and that only comes with having self-control. This view was exemplified by Operator 11, who stated the following:

Our confidence isn’t based on wishful thinking. It’s built brick by brick through preparation and endless hours of practice. When you’ve rehearsed a scenario a hundred times, you instinctively know how to respond, even under extreme stress.

Crucially, this quote highlights the commitment to continued practice, training, and development that is required, and the self-control needed to ensure that this preparation and practice always takes place.

Similarly, Operator 18 emphasized the role of discipline in the role:

Nobody passes without discipline. It takes that discipline to prepare beforehand, so you don’t rock up [turn up] on day one unprepared. You will only get so far on your natural fitness or talent, but sooner or later you’ll be found out [by the directing staff on selection]. That may come as lack of fitness on the hills [phase one of selection], or poor drills in the jungle [phase two of selection] but not having the discipline to diligently prepare will catch up with you.

### 4.9. Stubbornness

The next theme that emerged in this study related to being stubborn or steadfastly committed to a specific course of action. This stubbornness was reported as being important by several participants. For example, Operator 2, while discussing the characteristics of those who fail, suggested the following: “Guys who fail have a lack of confidence in their own abilities, and they don’t have that stubborn streak that would get them through otherwise”. Operator 13 reflected a similar view, stating the following: “the men who have that stubbornness, that ability to keep going even though you may think you’re not able to, or good enough to complete a task, those lads do well”. It is interesting to see the link here between the commitment to keep going even though there is doubt. This ability to keep going through hardship is a core component of the UK special forces selection process, selecting individuals who have this mindset, so it is possibly unsurprising this view permeates through those individuals who successfully pass selection.

### 4.10. Coping Mechanisms

The operators interviewed in this study also shared several strategies they used to manage stress, including pre-mission visualization, music, and mission debriefs. Operators said that they use music for relaxation or to “get in the zone” (Operator 14), and eight operators referred to the importance of squadron debriefs. An example of this perspective was presented by Operator 3, who highlighted the importance of those squadron debriefs to “keep the job where it happened, to be able to discuss and deal with stuff then move on”. This ability to be able to compartmentalize experiences seems to be important for remaining mentally healthy in the medium-to-long-term. Meanwhile, post-mission casual chats with peers were also highlighted as an important strategy to cope with stress. A point made by Operator 20, who suggested that these chats “helped to normalize the shit we had to do on ops”. This perspective highlights the importance of mutual sharing and social support with other operators who have been through similar experiences.

## 5. Discussion

**Figure 1:**
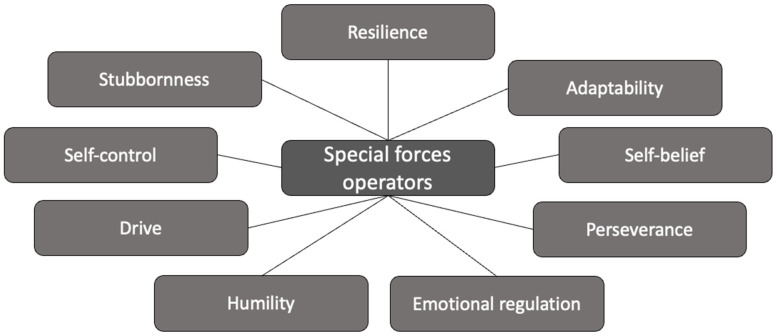
Psychological traits of UK special forces operators.

The aim of this research was to explore former special forces operators’ perceptions of the psychological traits required to be successful in the role of a special forces operator. The perceptions shared in this research suggest a distinctive psychological trait profile that is prevalent among UK special forces operators. This profile not only underpins their mental and behavioral tendencies but also serves as a foundation for their exceptional performance in the demanding roles they undertake. An overview of this profile is presented in Figure 1.

Resilience and adaptability were highlighted as crucial psychological traits in the current study. Unsurprisingly, these traits have also been reported in previous studies relating to the military. For example, both Nindl et al. (2018) and Bartone et al. (2008) highlighted the importance of resilience, hardiness, and adaptability in military professions. Resilience has also been identified as a contributory factor to successful selection into the special forces (Farina et al., 2019). It has been previously suggested that resilience in this context is a process, something that develops over time, culminating in its application in this context. Part of this development is through the training provided, but the experiential component, both in terms of early life experiences as well as operational experience, is also important (Crane et al., 2019).

Participants suggested that the deep-seated bonds of camaraderie and brotherhood that the soldiers shared offer an additional layer of support and resilience. The importance of the social environment has previously been highlighted as important for maintaining resilience in military personnel (Crane et al., 2019). This powerful sense of community and mutual trust likely acts as a buffer against the significant job-related stresses operators encounter. Consequently, the interaction between an individual’s psychological traits and the effect of their supportive environment appears to be a core building block in the development and maintenance of resilience in special forces operators.

Self-belief was another characteristic that was consistently highlighted by the participants in this study as being important for success, a characteristic also highlighted in other SOF-focused studies (e.g., Ytterbøl et al., 2022). Participants also outlined how this self-belief is crucial for both performance and selection into the service. This finding of the importance of self-belief is similar to Gruber et al. (2009), who reported a significant relationship between the associated factor of self-efficacy (belief in your ability to do something) and success in the selection process. In addition, participants in the current study reflected that their self-belief impacted upon their success, illuminating how self-belief helped drive them to push boundaries and redefine their own limits. The fact that the operators in this study said that it played such a pivotal role both during selection and throughout their careers further highlights the perceived importance of this trait. In contrast, self-doubt was highlighted by participants as a factor that negatively influenced their ability to be successful in their roles, with participants suggesting that a lack of self-belief was the downfall of many candidates during the selection phase. These reflections lend credence to the suggestion that strong self-belief is not just a desirable attribute but a crucial one for success (Bekesiene, 2023). The perceived connection outlined by these participants between failure in the special forces’ selection process and the absence of self-belief is a reminder of the power this singular trait is viewed to wield (Fosse et al., 2015).

The perceptions of a connection between self-belief and perseverance also became evident throughout the interviews conducted, a link supported in other military-focused studies (H. S. Meyer et al., 2018). This relationship between self-belief and perseverance has also been reported more widely in psychology. Specifically, the interaction between self-belief, self-efficacy (belief in one’s own abilities), and perseverance is presented in Social Cognitive Theory (Bandura, 1997). Participants also suggested that self-belief, perseverance, and emotional control were crucial to help special forces operators trust themselves, stay calm under stress, and keep going despite often facing significant challenges, a factor previously reported in U.S. Air Force (USAF) tactical operators (Ogle & Young, 2016).

Drive and motivational resources were also highlighted as important for special forces operators in the current study, factors that have previously been highlighted as important for special forces operators (De Beer & Heerden, 2014). Though, interestingly, previous research exploring motivation in a military context has highlighted the importance of leadership as a confounding variable in the relationship between motivation and performance (Siebold, 2012).

The notions of being “stubborn”, keeping going though you do not feel you can, and being stoically committed to a course of action were highlighted as important characteristics by participants in this study. While being stubborn might not normally be seen as positive, in the current study this trait was viewed as crucial for success. In many spheres of life, stubbornness might traditionally be perceived as a limitation, a sign of inflexibility, or a reluctance to embrace change (Harris et al., 2013). However, within the unique and demanding world of the special forces, stubbornness assumes a different, more positive connotation. In the challenging environments that special forces personnel operate in, this so-called stubbornness becomes an invaluable asset. Similar in this regard to the concept of perseverance, and in particular the persistence and cognitive flexibility characteristics implied (Credé et al., 2017). Stubbornness, as presented by the participants in this study, transforms into an unyielding determination, a relentless drive to see a task or mission through to its conclusion regardless of the obstacles. Indeed, previous research outside of the military context has reported a relationship between psychological stubbornness and resilience (Bordbar et al., 2022). This finding underlines the fact that the interpretation and value of certain psychological traits can be highly context dependent. What might be considered a liability in one setting could be an asset in another.

Emotional regulation was another key trait of participants in this study. This finding supports other military-focused research that has reported the importance of emotional regulation and control. For example, SOFs were reported by Skoglund et al. (2020) as scoring higher on emotional stability than non-specialist military forces. The development of emotional regulation skills has been seen as crucial to developing more effective and resilient soldiers (Algoe & Fredrickson, 2011). Indeed, a lack of emotional regulation has also previously been reported as an issue within the US armed forces (Stanley & Larsen, 2021).

The identification by participants in this study of humility as a crucial trait offers an interesting insight into better understanding the nature of successful UK special forces operators. Traditionally, the popular image of special forces operators, as shaped by various media portrayals and public perceptions, often leans heavily towards characteristics like unyielding confidence, perhaps even bordering on arrogance (Lamb, 2003). However, this study’s findings suggest a counter-narrative that prioritizes the trait of humility. Such humility underscores the willingness and eagerness of these operators to be perpetual learners, open to feedback and continuously evolving in their roles (Gayton & Kehoe, 2016). This view highlights that while self-belief is undoubtedly valuable, the ability to be self-reflective, acknowledge one’s limitations, and actively seek growth opportunities is equally, if not more, important (Crane et al., 2019). This nuanced perspective challenges and broadens the more simplistic and potentially misleading stereotype of the hard-nosed, individualistic operator, painting a more holistic picture of the depth and diversity of traits that truly define special forces operators. At face value it might seem odd that both humility and stubbornness were cited as important. But in the current study the traits appear to balance one another. With stubbornness more closely linked to the concept of perseverance (adaptability, focus, and motivation) and humility (receptivity to feedback, other-oriented focus, and accurate self-assessment) (Wright et al., 2016).

Another trait highlighted in the current study as important was self-control. Seen by participants as important for effective performance and ongoing success for SOFs. Self-control has previously been highlighted as an important factor for military personnel, particularly when it comes to the evaluation of risks, decision-making, and ultimately risk-taking behavior (Sicard et al., 2001). Self-control was also reported to be an important character strength in US Air Force officers (Sosik et al., 2018).

An important factor that emerged from the interviews with participants in the current study related to their coping mechanisms. Specifically, the strategies they employ to mitigate the stressors inherent in their operator roles. A prominent strategy shared by participants was pre-mission visualization. Several participants highlighted the value of mentally rehearsing the mission beforehand, a technique previously suggested as important for military populations (V. M. Meyer, 2018; Blacker et al., 2019; Jensen et al., 2020). This approach likely provides the operators with a sense of control and preparation, allowing them to mentally traverse potential scenarios, thereby enhancing their readiness to respond to real-time challenges. Music was also suggested to be an important strategy to cope with the challenges of the job, with the effect dependent upon the specific traits of the individual operator. Several participants highlighted music as an important tool that enabled them to both cope with and gain separation from the demands of the role. This outcome supports the extensive literature highlighting the positive psychological impact of music for stress management and mood enhancement (Gooding & Langston, 2019; Zoteyva et al., 2015). Music has the potential to create a mental space where operators can mentally distance themselves momentarily from immediate stresses, recalibrate, and re-engage with renewed vigor. It might also serve as a bridge to more pleasant memories or feelings, offering a brief respite from the demanding present. Other factors that participants outlined as enhancing their ability to cope included the formal debriefs that take place and conversations with peers. Indeed, the significance of squadron debriefs and post-mission casual conversations with peers cannot be understated. According to participants in this study, these interactions serve a dual purpose. Squadron debriefs ensure that operational learning is captured, but they also offer a structured space to process events, which can be crucial for psychological well-being (Lin et al., 2020). Casual peer chats, on the other hand, provide an informal, unstructured environment to debrief emotionally, ensuring that operators do not bottle up emotions and can share experiences, challenges, or doubts. Evidence from other domains suggests that immediate debriefs after traumatic events can be effective in enhancing wellbeing (Johnson et al., 2021). One potential limitation of the current study was the fact that participants were retired from service, rather than current special forces operators. This could potentially result in a dated view of the demands of the role. It was felt that requiring participants to have served within the last five years served as an effective strategy to limit this potential effect.

## 6. Conclusions

The results in this study suggest a distinctive psychological trait profile for UK SOFs when compared to other special forces populations. This UK SOF profile is composed of some traits previously reported in research with SOFs from different countries, including resilience, adaptability, self-belief, perseverance, emotional regulation, drive, and self-control. With the traits of humility and stubbornness uniquely reported for this UK population. These traits were suggested to be the foundation for their performance in the high-stress and volatile environments they perform in. These traits are not merely a list of isolated qualities but appear to be inter-related, each influencing the other, and together creating a robust framework that equips these individuals for the challenges they confront.

Another important outcome was the highlighting of the essential role of the supportive environment, particularly the bonds of camaraderie and brotherhood, in enhancing resilience. The study suggests that while individual attributes are critical, the presence of a supportive environment is crucial for continued success.

While this research is a start, further research is needed to develop a deeper understanding of the psychological traits underpinning longevity and performance. Crucially, understanding the degree to which these traits can be developed or trained. These findings can also help to underpin the development of special forces-specific programs of support and development.

## Data Availability

The participants of this study did not give written consent for their data to be shared publicly, so, due to the sensitive nature of the research, supporting data are not available.
